# The Identification of a Sub-Micromolar Peptide-Based Protein Arginine Methyltransferase 1 (PRMT1) Inhibitor from a Plate-Based Screening Assay

**DOI:** 10.3390/biom15111494

**Published:** 2025-10-23

**Authors:** Tina M. Sawatzky, Sarah A. Mann, Jordan Shauna Tucker, Aida A. Bibart, Corey P. Causey, Bryan Knuckley

**Affiliations:** Department of Chemistry & Biochemistry, University of North Florida, Jacksonville, FL 32224, USA; tmariesawatzky@gmail.com (T.M.S.); mannsc@bc.edu (S.A.M.); artistjst@hotmail.com (J.S.T.); aabibart@ufl.edu (A.A.B.); c.causey.129741@unf.edu (C.P.C.)

**Keywords:** PRMT, arginine, methyltransferase, substrate specificity, histones, post-translational, enzyme, inhibitor, screening assay, protein arginine methyltransferase

## Abstract

Post-translational modifications (PTMs) expand the structural diversity of proteins beyond the standard amino acids, influencing protein-protein interactions. Protein methylation, a prevalent PTM, involves the transfer of methyl groups from S-adenosylmethionine (SAM) to lysine and arginine residues. Arginine methylation is catalyzed by the Protein Arginine Methyltransferase (PRMT) family to yield mono- and dimethylarginine forms. PRMT1, the isozyme responsible for the majority of asymmetric dimethylation (ADMA) is implicated in various diseases, including cancer. Here, we report the synthesis and screening of a second-generation peptide library to identify novel PRMT1 substrates. The library, based on histone peptides, incorporated varying sequences of amino acids, facilitating substrate specificity studies. Screening identified 7 peptide sequences as exceptional PRMT1 substrates, which were confirmed by kinetic analysis. Consensus sequences revealed key recognition elements for PRMT1 catalysis, suggesting roles for small non-polar side chains and specific residues near the substrate arginine. Furthermore, we developed a peptide-based PRMT1 inhibitor by substituting the substrate arginine with a chloroacetamidine warhead. The inhibitor exhibited sub-micromolar inhibitory potency against PRMT1, surpassing previous peptide-based inhibitors. Our findings contribute to understanding PRMT1 substrate specificity and provide a scaffold for developing potent inhibitors targeting PRMT1 in diseases, including cancer.

## 1. Introduction

Post-translational modifications (PTMs) have long been recognized for imparting additional structural elements to proteins beyond those found in the 20 proteinaceous amino acids. These modifications, which occur on the side chains of residues, can result in altered interactions between the modified protein and its binding partners. Protein methylation, which involves the transfer of a methyl group from S-adenosylmethionine (SAM) to the side chain of lysine, histidine, and arginine residues, is one of the most widely studied PTMs. These modifications can include the transfer of multiple methyl groups and, in the case of arginine, can result in monomethylated, symmetric dimethylated, and asymmetric dimethylated guanidium groups [1,2,3].

**Figure 1:**
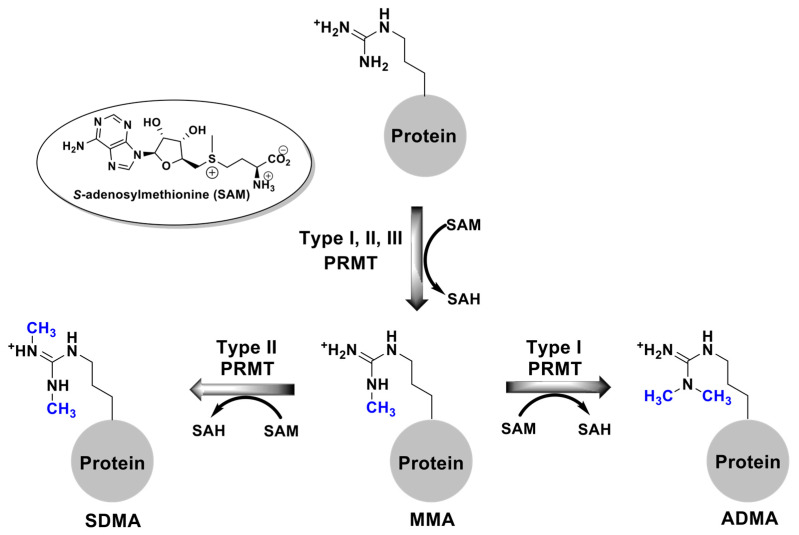
The reaction catalyzed by the Protein Arginine Methyltransferase family of enzymes (PRMT). Methyl groups are transferred from S-adenosylmethionine (SAM) to a terminal nitrogen of protein-arginine resulting in asymmetric dimethylarginine (ADMA; Type I PRMT), symmetric dimethylarginine (SDMA; Type II PRMT), or monomethylated arginine (MMA; Type III PRMT).

The transfer of methyl groups from SAM to arginine residues is catalyzed by a family of enzymes known as the Protein Arginine Methyltransferases (PRMTs). The eleven members of this family are subcategorized into three different types. Type I PRMTs (1, 2, 3, 4, 6, 8, 10, and 11) produce asymmetric dimethyl arginine (ADMA) residues. The asymmetry occurs because both methyl groups are transferred to one of the terminal nitrogens of the guanidinium moiety. Type II PRMTs (5 and 9) produce symmetric dimethyl arginine (SDMA) residues, where the symmetry results from one methyl group being transferred to each of the terminal nitrogens of the guanidinium group. The lone Type III isozyme (PRMT 7) catalyzes the transfer of a single methyl group, yielding monomethylarginine (MMA) residues (Figure 1) [1,2,3,4]. These structural alterations on the guanidinium group change the properties of the moiety both sterically and through their hydrogen bonding abilities [5,6,7].

The biological response to arginine methylation is dependent both on the location and the type of modification (i.e., ADMA versus SDMA). One well known substrate group for PRMTs are the N-terminal tails of histone proteins. Histones are a constituent part of chromatin structure—four histone proteins (H2A, H2B, H3, and H4) form dimers of heterotetramers around which DNA strands are wrapped to form nucleosomes. The N-terminal tails of these proteins protrude beyond the core of the compacted structure and often undergo post-translational modifications—these modifications affect the expression of certain gene products [5,8]. Given this effect on gene expression, arginine methylation has been associated with multiple diseases, thereby suggesting PRMTs as potential targets for therapeutic intervention [1,7]. For example, when Arg-3 of the histone H4 (H4R3) is methylated by PRMT1, the acetyltransferase p300 is recruited to make further modifications that can activate the tumor specific androgen receptor (AR) target genes. The AR is a nuclear receptor that assists in regulating gene expression, and overexpression can lead to prostate cancer initiation and progression [9]. In another notable example, the asymmetric dimethylation of Arg-8 of histone H3 (H3R8) by PRMT2 acts as a coactivator in glioblastoma multiform progression [10]. Given these correlations, the development of isozyme-specific inhibitors could allow attenuation of diseases.

The most abundant isozyme in eukaryotic cells is PRMT1, and this enzyme is responsible for the majority of asymmetric dimethylation [11]. Modification of the Arg-3 residue of histone H4 (H4R3) by PRMT1 leads to pre-mRNA splicing and DNA signaling, which has been linked to cancers of the breast, colon, bladder, lung, and blood [12,13,14,15,16]. Given its putative role in these diseases, the development of a specific inhibitor for this isozyme could have significant medical implications. Towards this goal, much work has been conducted to understand the substrate specificity among the isozymes [17,18,19,20,21]. We previously reported a novel plate-based assay to gain insights into peptide substrate specificity [22]. Results from this assay were used to develop a novel peptide-based inhibitor with demonstrated selectivity for PRMT1 over PRMT5 [23]. This substrate sequence information was then used to develop a peptoid-based inhibitor that initiates cell death in both MDA468 and HCT116 cancer cells, which suggests PRMT1 as a viable therapeutic target and thus the development of more potent inhibitors is therefore warranted [24,25]. Herein we report the synthesis and screening of a second-generation peptide library and the identification of a new peptide-based inhibitor with sub-micromolar inhibitory potency against PRMT1.

## 2. Materials and Methods

Reagents for solid-phase peptide synthesis were purchased from Peptides International (Louisville, KY, USA), Chem-Impex Int’l Inc. (Wood Dale, IL, USA), AnaSpec Inc. (Freemont, CA, USA), Tokyo Chemical Industry Co., Ltd. (Tokyo, Japan), Oakwood Chemical (Estill, SC, USA), NovaBiochem (Darmstadt, Germany), Alfa Aesar (Ward Mill, MA, USA) and Fisher Chemical (Fair Lawn, NJ, USA). The plate washing vacuum manifold was a product of Enzo Life Sciences (Farmingdale, NY, USA). The MTAse-Glo Methyltransferase Assay (Cat#V7601) was acquired from Promega (Madison, WI, USA). PRMT1 was expressed and purified as previously described [22].

### 2.1. Synthesis of H4-11R Peptide

The Conserved Region Peptide sequence of NH_2_-RGGKGLGKGGAK-COO^−^ was synthesized using Fmoc solid-phase peptide synthesis on 320 µm TentaGel Macrobead-NH_2_ resin (0.25 mmol/g; 130 mg). A C-terminus Pro-Asp linker was incorporated to provide cleavage of the peptide from the resin using 49.5% Acetonitrile: 49.5% H_2_O + 1.0% Formic Acid. The resin was incubated with seven equivalents of both HBTU and the Fmoc-protected amino acid with 5% *N*-methylmorpholine (NMM) dissolved in 3 mL dimethylformamide (DMF). Coupling was confirmed using the ninhydrin test on a small amount of resin beads. The synthesis and sequence of the completed peptide was confirmed by electrospray ionization mass spectrometry (ESI-MS).

### 2.2. Synthesis of 96 Variable Peptides in Multi-Well Plate

The H4-11R peptide beads were divided evenly into a Pall Corporation AcroPrep Advance 96-well filter plate (Cat#8047; Pall Corporation; Port Washington, NY, USA) resulting in a 1.025 × 10^−4^ mmol scale in each well (~20 beads/well). The remaining variable amino acid residues were added to individual wells by incubating the beads in each well with 5 equivalents of the amino acid, 5 equivalents of HBTU, 5% NMM in 200 μL of DMF for 120 min with gentle rocking. The individual wells were washed with DMF (5 × 5 mL). The Fmoc groups were removed by treating all wells with 20% piperidine in DMF (200 μL) twice for 15 min. The wells were washed again with DMF (5 × 5 mL). This process was repeated for each of the variable positions of the peptide to create 96 different peptides to be utilized in the screening protocol. Three wells of the 96-well plate were designated as controls to verify assay performance. In two of these wells, a PRMT1 control substrate (Linker Region–Conserved Region–GGRGG) was synthesized to serve as positive and negative controls (+/−PRMT1), while the third well contained a positive control incorporating asymmetric dimethylarginine (Linker Region–Conserved Region–GG-ADMA-GG). Beads were first treated with 95% TFA: 2.5% TIS: 2.5% H_2_O to cleave side chain protecting group before cleaving the peptide from the resin using 49.5% acetonitrile: 49.5% H_2_O + 1.0% formic acid and heating at 95 °C for 25 min. To validate, the expected mass was compared to the observed mass using Thermo Fisher Scientific LTQ XL ESI-MS (Supplemental Table S1; Waltham, MA, USA).

### 2.3. Screening the Substrate Plate with PRMT1

The beads from the filter plate were transferred to an Acrowell 96 Filter screening plate (Cat#5020; Pall Corporation; Port Washington, NY) and rinsed with 0.20 mL DI water, followed by 0.20 mL of TBST 10 times. The beads were swelled with 0.25 mL TBST overnight with gentle rocking at 4 °C. The plate was rinsed with 0.25 mL of Assay Buffer #1 (50 mM HEPES pH 8.0, 50 mM NaCl, 1 mM EDTA). After draining, the plate was incubated with 500 nM PRMT1 in 0.1 mL of Assay Buffer #2 (50 mM HEPES pH 8.0, 50 mM NaCl, 1 mM EDTA, 0.5 mM DTT, 400 µM SAM) for 90 min at 37 °C while rocked gently at 120 rpm. The plate was rinsed with TBST (5 × 0.20 mL) then rocked gently in 100 µL of 0.1% SDS for 15 min at room temperature, before being washed with DI water (10 × 0.20 mL) and TBST (10 × 0.20 mL).

Beads were blocked with 125 µL of 1.5% (*w*/*v*) BSA in TBST for 1 h at room temperature with gentle rocking. The beads were rinsed with TBST (5 × 0.20 mL), then incubated overnight at 4 °C with 0.065 mL total volume of TBST + 1.5% (*w*/*v*) BSA + 1:500 Ab412 antibody (purchased from Abcam; Cambridge, UK) with gentle rocking.

The plate was then rinsed with 0.20 mL of TBS for 10 min and 0.20 mL TBST for another 10 min. The plate was incubated with gentle rocking for 60 min at room temperature with 0.065 mL total volume of TBST + 1.5% (*w*/*v*) BSA + 1:4000 Ab98690 antibody (secondary antibody: purchased from Abcam; Cambridge, UK). This mixture was rinsed with TBS (10 × 0.20 mL), then 0.2 mL TBST for 10 min. The plate was then incubated with 2.5 mg/mL 5-Bromo-4-chloro-3-indolyl phosphate (BCIP: a substrate that detects alkaline phosphatase emitting a blue hue when conjugated with secondary antibodies) dissolved in BCIP buffer (5.85 g NaCl, 12.1 g Tris-base, 0.476 g MgCl_2_ in 1 L of DI water, pH 9.0) at room temperature for 1 h with gentle rocking. The reaction was quenched with 0.1 M HCl. The beads were examined under a microscope to determine if the majority of beads in a well displayed a deep blue hue, and these were determined to be potential substrates of PRMT1.

### 2.4. Synthesis and Purification of Individual Peptides

Peptides were synthesized in a 6.0 mL Teflon Tyvek syringe (Torviq; Tuscon, AZ, USA) on a 0.40 mmol scale of Rink Amide MBHA resin (50 mg). Synthesis was completed similar to that described previously for the H4-11R peptide using standard Fmoc solid-phase peptide synthesis. The completed peptides were cleaved from the resin in 95% trifluoroacetic acid (TFA), 2.5% triisopropylsilane (TIS) and 2.5% H_2_O (total volume 2.5 mL), rocking gently at room temperature for 30 min. The TFA was evaporated, and the remaining solution was treated with cold diethyl ether to precipitate the peptide. The crude samples were purified using reverse-phase HPLC (Agilent Technologies, Santa Clara, CA, USA), the final masses were confirmed by ESI-MS (Supplemental Table S2), and the samples were lyophilized to yield the peptide products.

### 2.5. Synthesis of Chloroacetamidine Warhead Peptide

A peptide with the sequence of COO^−^KAGGKGLGKGG-Orn-AKCK-AcLys-NH_2_. (Orn standing for ornithine and AcLys for acetylated lysine) was synthesized using Fmoc solid-phase peptide synthesis on 50 mg of Rink amide MBHA resin in a 5.0 mL Teflon Tyvek syringe as previously discussed. Once the sequence was complete, the terminal Fmoc group was removed, and the mass of the peptide was confirmed via MS before addition of the chloroacetamidine warhead. The beads were treated with 10 equivalents of Boc anhydride (di-tert-butyl-di-carbonate) in 5% NMM in DMF (3 mL total volume) and rocked gently for 60 min. The beads were rinsed 5 times with DMF and 5 times with CH_2_Cl_2_. Addition of the Boc group was confirmed with the ninhydrin test. The DDE protecting group was removed from the ornithine residue by adding 5 mL of a 2% hydrazine in DMF solution to the syringe and rocking it for 45 min at room temperature. The removal of the DDE protecting group was confirmed with the ninhydrin test. Ethyl chloroacetimidate (6 equiv., 50 mg) was dissolved in 3 mL of dry DMF and this solution was then added into the syringe. Triethylamine (9 equiv., 87 µL) was added, and the syringe was rocked for 24 h at room temperature. The addition of this chloroacetamidine warhead was confirmed via ESI-MS (Supplemental Table S2).

### 2.6. Determining Kinetic Parameters of Validation Peptides

To determine the kinetic parameters, *k_cat_*, *K_m_* and *k_cat_*/*K_m_* for each of the validation or hit peptides, a MTase-Glo™ Methyltransferase Assay from the Promega Corporation was utilized to monitor the enzymatic reaction.

Varying concentrations (0–1000 µM) of the peptide in assay buffer (4 mM DTT, 0.4 mg/mL BSA, 4 mM EDTA, 12 mM MgCl_2_, 200 mM NaCl and 80 mM Tris Buffer-pH 8.0) were incubated at 37 °C for 10 min. Then 4 µL of 200 nM PRMT1 was added to this reaction mixture and continued for another 10 min at 37 °C. Of note the enzymatic activity was linear with respect to time and enzyme concentration under these conditions. The reaction was quenched by the addition of 2 µL 0.5% TFA and placed at room temperature before adding 2.2 µL of the 6× MTase-Glo Reagent. After 30 min, 13.2 µL of the MTase-Glo Detection Solution was added and incubated for an additional 30 min before the luminescence was read using a BioTek Synergy 2 Multi-Mode Microplate Reader (Agilent Technologies, Santa Clara, CA). Luminescence signal was converted to product concentration using SAH standards (0–5 μM) and the initial rates were fit to Equation (1):

V_o_ = (*K_m_* + [S]/Vmax [S]) (1)

using GraFit version 7.03.

### 2.7. Determining Inhibition Parameters of Chloroacetamidine Warhead Peptide

The MTase-Glo assay was also used to determine the inhibition parameters of the chloroacetamidine warhead peptide. For this assay, various concentrations of the chloroacetamidine warhead peptide (0–250 µM) were added to reaction buffer (80 mM Tris Buffer [pH 8.0], 200 nM NaCl, 4 mM EDTA, 12 mM MgCl_2_, 0.4 mg/mL BSA and 4 mM DTT) and incubated for 10 min at 37 °C. Following this incubation, 4 µL of 400 nM PRMT1 was added to the inhibition reaction mix and continued to be incubated at 37 °C for 15 min. The substrate peptide, 1 uL of 225 µM AcH4-21, was added to each reaction for 15 min. To quench the reaction, each sample received 2 µL of 0.5% TFA solution and placed at room temperature before 2.2 µL of the 6× MTase-Glo reagent was added to each sample for 30 min. Then 13.2 µL of the MTase-Glo Detection solution was added and incubated for an additional 30 min. The luminescence signal was read using a BioTek Synergy 2 Multi-Mode Microplate Reader and the % activity was calculated using an SAH standard curve (0–5 µM) before being fit to Equation (2):

(2) $$Fractional\;Activity\;of\;PRMT=\frac{1}{\left(1+\;\frac{1}{{IC}_{50}}\right)}$$

using GraFit version 7.03.

## 3. Results

**Design and Synthesis of the Peptide Library**. Histone peptides are well-documented substrates of PRMT1 and thus provide a good model for the design of a screening library to search for better PRMT1 peptide substrates. More specifically, the Histone H4-16 peptide has a *k_cat_*/*K_m_* value of 1.90 × 10^3^ M^−1^ min^−1^ and is recognized as a mediocre substrate for the enzyme [22]. Given that our study is focused on identifying peptide substrates with improved kinetic parameters, we chose to use a library based on the H4-16 template. Our previous work highlighted that flexibility was permitted within the first five residues from the N-terminus, while preserving the next 11 residues was important. These 11 amino acids, residues 6–16 of H4-16, NH_2_-GGKGLGKGGAK-COO^−^, provide a distal positive charge from the Arg being modified, which has been reported as a crucial factor for substrate binding [26]. However, it has also been found that the substrate arginine residue can be sandwiched between this conserved 11-residue sequence and a varied first five amino acids (residues 1–5) [22]. Information about advantageous substitutions around the arginine will reveal specific structural features that may yield better inhibitors.

**Figure 2:**
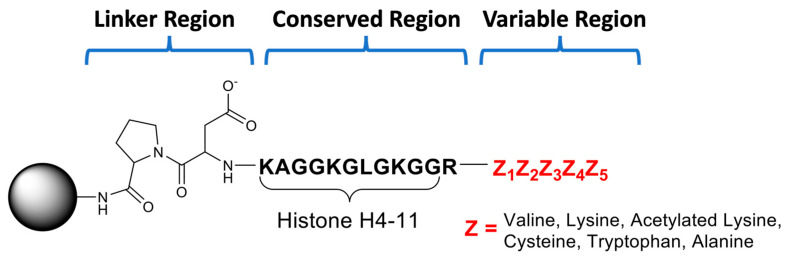
The general structure of the peptide library beads making up the one-bead one-compound library. The beads consisted of a Pro-Asp cleavable linker region followed by the first 11 residues of Histone H4 plus the substrate Arg residue (Conserved Region). A variable pentamer region consisting of 6 amino acids (Val, Lys, AcLys, Cys, Trp, and Ala) terminated the library beads (Variable Region). A total of 96 different peptides were synthesized in a 96-well filter plate using standard Fmoc synthesis.

To develop the second-generation library, we utilized standard Fmoc solid-phase peptide synthesis. Peptides were built onto resin beads using a Pro-Asp linker that allows easy cleavage of the peptide from the resin. This linker was followed by residues 6–16 of H4-16 (NH_2_-GGKGLGKGGAK-COO^−^; Conserved Region; Residues 7–17), the substrate arginine (Residue 6), and five variable residues (Variable Region; Residues 1–5). For the variable region, we incorporated 6 amino acids (Cys, Ala, Trp, Val, Lys, and AcLys) at 5 randomized positions (Figure 2). Each of these amino acids were chosen because they are either typically found in histones (Ala and Lys) or they are non-traditional amino acid residues that provide alterations in charge, size, or polarity. Additionally, we limited the number of amino acids to keep the library small and focused, including only six amino acids in total. Though many amino acids could have been considered, we restricted our selection to those most interesting for altering charge, polarity, or size.

The library was constructed by dividing beads containing the Linker + Conserved Regions into a 96-well filter plate with approximately 20 beads/well. The variable peptide portion of the sequences was then added to these beads by randomly assigning additional residues from our chosen 6 amino acids. To do this, we added the Fmoc solid-phase peptide reagents directly to each well, generating 93 random peptides with 20 identical beads/well (Supplemental Table S3). The other three wells of the 96-well plate contained a set of controls: a positive peptide control incorporating asymmetric dimethylarginine and two wells with a PRMT1 control substrate (Linker Region–Conserved Regions–GGRGG sequence) that would be incubated in the presence or absence of PRMT1 during the screen. Beads from random wells of the multi-well plate were chosen and cleaved from the resin and analyzed by ESI-MS/MS to confirm the peptide sequence (Supplemental Table S1).

**Screening the Peptide Library with PRMT1**. The ability to identify novel peptide sequences as substrates for PRMT1 provides insight into the substrate specificity of the active site, which can lead to the development of potent and selective inhibitors of this enzyme. Typical screening methods for identifying enzyme substrates can be time-consuming and laborious, so we previously developed a substrate screen using a multi-well plate-based methodology that recognizes novel sequences from a peptide library that have been methylated by PRMT1 [22].

**Figure 3:**
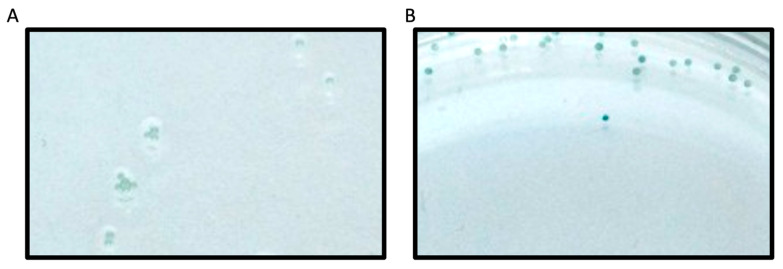
Microscopy images of the PRMT1 control substrate as (**A**) a negative control, without PRMT1, and (**B**) a positive control, with PRMT1. The alkaline phosphatase substrate, BCIP, generates a blue precipitate on beads displaying asymmetric dimethylarginine (ADMA), illustrating the observed difference between ‘hits’ and ‘non-hits.

**Figure 4:**
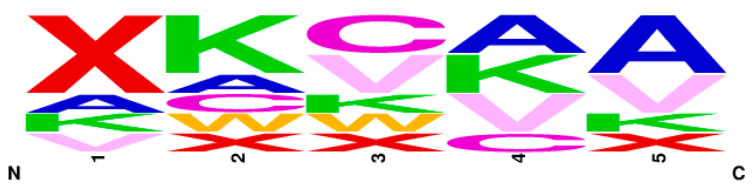
The consensus sequence determined from the seven hit peptide sequences based on the frequency of the amino acids at each position. Note the Linker is attached on the C-terminus. The variable region (5 residues) of the peptide contained combinations of X = Acetylated Lysine, W = Tryptophan, C = Cysteine, V = Valine, K = Lysine, A = Alanine.

Previously, we reported on this novel plate-based screening assay of the PRMT family of enzymes and identified a PRMT1 peptide substrate with *k_cat_*/*K_m_* of 8.1 × 10^4^ M^−1^ min^−1^, which validated this screening methodology [23]. In the current work, we utilized this method to screen a second-generation peptide library against PRMT1, which incorporated amino acids with diverse charge, size, and PTMs around the substrate arginine. Beads functionalized with peptide library members were washed extensively with buffers and subsequently incubated with PRMT1 for 60 min. The methylation reaction was then quenched with sodium dodecyl sulfate to denature PRMT1 and halt the enzymatic reaction. The peptide beads that contained ADMA as a result of PRMT1 action were identified using a 1° antibody that binds only to ADMA. Given our screen was focused on identifying only the very best substrates, we were not concerned with peptides that may have displayed MMA. The beads were then incubated with a 2° antibody conjugated to alkaline phosphatase. The alkaline phosphatase deposits a blue precipitate on the beads upon treatment with alkaline phosphatase substrate (BCIP; Figure 3). Thus, the blue precipitate would only be produced on beads that displayed ADMA, which would allow us to easily identify the sequences that were PRMT1 substrates. It is important to note that each well contained 20 beads that all displayed the same peptide sequence. This redundancy allowed us to reduce false positives and improve the reliability of our screening assay. Any wells that contained at least 10 blue beads were chosen as “hits”. In total, 7 of the 93 wells met these criteria and were chosen for further validation studies (Table 1). In addition, a series of consensus sequences was identified from the 7 hit sequences using the WebLogo generator (https://weblogo.berkeley.edu/logo.cgi (accessed on 27 August 2021); Figure 4).

**Validation of Hit Peptides and Consensus Sequence Peptides**. To validate the results of the screen, we synthesized and purified two of the “hit” peptide sequences (Wells D5 and G2) and two “non-hit” peptide sequences (Wells B10 and E9) before measuring their kinetic parameters (*k_cat_* and *K_m_*) using a standard Methyltransferase Luminescence Assay (Table 2; MTase Glo, Promega). The *k_cat_* and *K_m_* values for the “hit” Well D5 were 1.912 ± 0.113 min^−1^ and 6.78 ± 1.77 μM (*k_cat_*/*K_m_* = 2.821 × 10^5^ M^−1^ min^−1^), respectively. The *k_cat_* and *K_m_* values for the “hit” Well G2 were 0.986 ± 0.004 min^−1^ and 25.4 ± 3.95 μM (*k_cat_*/*K_m_* = 3.89 × 10^4^ M^−1^ min^−1^), respectively (Supplemental Figure S1). The *k_cat_* and *K_m_* values for the “non-hit” Wells B10 and E9 were not detectable at concentrations of substrate up to 500 μM (Supplemental Figure S2). Based on this information, we were confident that our screen worked as intended and could identify substrates of PRMT1.

Rather than testing all individual hit peptide sequences, and to help identify the specific elements that are important for PRMT1 substrate binding, we collected and synthesized a series of consensus-based sequences from the list of hit peptides. Based on this data, we synthesized the top 4 consensus sequences (VALPep 1–4) and tested them using the MTase Glo Assay to determine their kinetic parameters (Table 3 and Supplemental Figure S3). Interestingly, but not unexpectedly, all 4 showed significant kinetic activity with *k_cat_* values ranging from 0.927–2.576 min^−1^ and *K_m_* values ranging from 6.92–8.72 μM and resulting in *k_cat_*/*K_m_* values of ~10^5^ M^−1^ min^−1^. These values are all greater than the *k_cat_*/*K_m_* value for Histone H4-16 and would be equivalent to the H4-21 peptide (*k_cat_*/*K_m_* = 2.80 × 10^5^ M^−1^ min^−1^), thus all the validation consensus peptides are excellent substrates of PRMT1.

**Figure 5:**
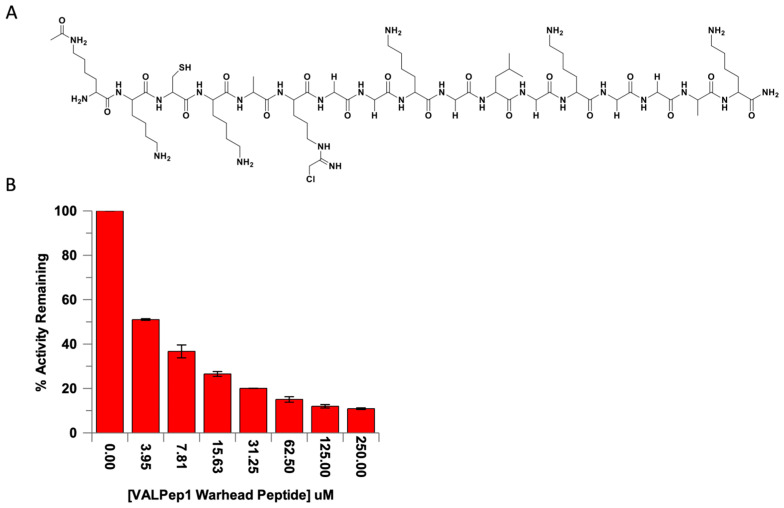
(**A**) The structure and sequence of the VALPep1 Warhead Peptide containing a chloroacetamidine warhead that replaces the guanidinium on the Arg side chain of the VALPep1 consensus sequence peptide (NH_2_-XKCKARGGKGLGKGGAK-COOH). (**B**) The IC_50_ plot of VALPep1 Warhead Peptide shows it inhibits PRMT1 at low µM concentrations.

**Development and Analysis of a PRMT1 Inhibitor containing a Chloroacetamidine Warhead.** Based on the consensus sequence data and their corresponding kinetic parameters, it was evident that the assay described herein was able to identify an excellent peptide substrate that bound strongly to the enzyme. The identification of novel peptide sequences, especially those that vary around the PRMT1 active site, provides a useful scaffold for inhibitor development. For that reason, the peptide sequence was converted to a PRMT1 inhibitor by substituting the guanidinium group of Arg with a chloroacetamidine warhead, which has been previously shown to covalently modify Cys101 in the PRMT1 active site (Figure 5A) [27]. Specifically, we chose the top consensus sequence identified in our screen, VALPep1, to create a novel PRMT1 inhibitor. The peptide was synthesized, and the warhead was installed in place of the guanidinium group of Arg (position 6 of the sequence). Ultimately, we measured the IC_50_ of this VALPep1 Warhead Peptide by incubating it in the presence of PRMT1 (Figure 5B). The IC_50_ value was 3.47 ± 0.46 μM, and this value is better than previously identified peptide-based inhibitors of PRMT1 [23,24].

## 4. Discussion

Post-translational modification of histone proteins by PRMTs has been shown to activate and repress cancer-related genes [12,13,14,15,16,28]. In addition, these enzymes are found in both the nucleus and cytoplasm, resulting in the methylation of many cellular proteins. The dysregulation of PRMTs has been identified as a key factor in cancer and other diseases, such as cardiovascular disease, viral pathogenesis, and spinal muscular atrophy [29]. Learning more about the key factors that drive substrate recognition and catalysis by PRMTs can aid in the development of potent and selective PRMT inhibitors that help to eliminate or reduce disease severity. We sought to alleviate the bottleneck in this process by utilizing a medium-throughput screening platform for the rapid identification of key residues that contribute to the substrate specificity of PRMT1. Previously, we screened a histone-based peptide library using this methodology to identify a novel peptide substrate with a *k_cat_*/*K_m_* = 8.1 × 10^4^ M^−1^ min^−1^ [22]. Given the success of that study, we decided to expand and develop a second-generation peptide library that incorporated amino acids typically found in histones (Ala and Lys), as well as some non-traditional amino acid residues (Val, Trp, Cys, and Acetyl-Lys). The library was designed by varying these 6 amino acids in the first 5 positions from the N-terminus and coupling it to Arg (substrate residue) followed by residues 6–16 of H4-16, NH_2_-GGKGLGKGGAK-COO^−^. This design allowed us to retain some of the distal positive charge that is important for PRMT1 substrate binding [26], while providing some flexibility with residues in or near the active site. In total, we synthesized 93 different peptides, which were displayed on beads in a 96-well plate. The peptides were screened as PRMT1 substrates using the previously developed substrate specificity screening assay and 7 novel peptide sequences were identified as substrates for this enzyme. Given this information, we looked to identify consensus sequences from the 7 hit beads to provide us additional information regarding specific recognition elements that were important for substrate binding and catalysis by PRMT1. The screening assay we implemented is reliable and provides a rapid method for synthesizing and screening hundreds of peptides as PRMT1 substrates. Using hit sequences from our screen, we generated a consensus sequence and synthesized consensus sequence-derived peptides to determine if we could identify specific recognition elements that would make these excellent PRMT1 substrates. The top four consensus sequences resulted in *k_cat_*/*K_m_* values of ~10^5^ M^−1^ min^−1^. Furthermore, one of the best-known PRMT1 peptide substrates is Histone H4-21 with a *k_cat_*/*K_m_* = 2.8 × 10^5^ M^−1^ min^−1^. Our assay screened only 93 peptides and resulted in identifying a substrate with kinetic parameters similar to those of H4-21 even though it is four residues shorter. Interestingly, the *K_m_* for VALPeps 1–4 decreased by ~20-fold as compared to Histone H4-16 peptide, whereas the *k_cat_* values increased by at least 3-fold. This finding suggests the consensus peptides bind more efficiently to PRMT1 but may be catalyzed a bit slower. Based on the design of our screening assay, this would make sense as binding is the most important factor in selecting potential substrates in our assay. We incubate the enzyme with beads for 90 min, thus substrates can be bound (*K_m_* effect), whereas the rate of the reaction may be much slower but still proceed (*k_cat_* effect).

**Figure 6:**
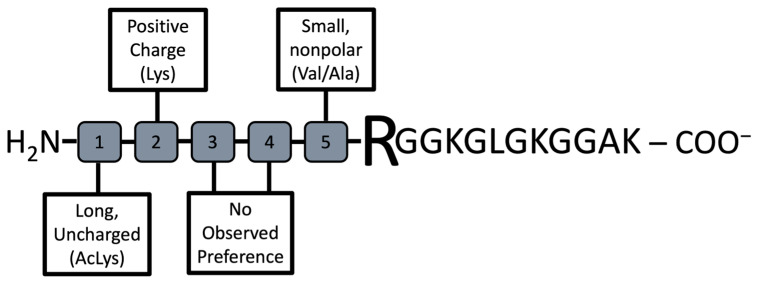
Preferred recognition elements for PRMT1 near the substrate Arginine residue.

Our goal was to identify preferred elements that promoted substrate binding by PRMT1 (Figure 6). Reviewing the data suggests that small non-polar side chains (Ala or Val) directly adjacent to the substrate Arg support binding. This same result is observed with Hit G2, but not in the non-hit compounds tested. These results agree with previous studies highlighting that peptides comprising of RGG motifs are good PRMT1 substrates [6,30]. Furthermore, this study identified that long, uncharged residues at position #1 of the N-terminus of the peptide (5 residues from the Arg substrate) are an important factor for PRMT1 substrate binding and catalysis. All of the consensus sequence peptides contained an acetylated-Lys at this position, which led to a significant improvement in catalysis for these peptides over Histone H4-16. In total, half of our hits contained an acetylated-Lys at position #1, whereas the non-hit peptides tested did not contain this residue. Lastly, the positive charge found at position #2 (4 residues toward the N-terminus from the substrate Arg) of the consensus sequence peptides improved binding. This residue was conserved in three of the hit peptides but was not present in the non-hit peptides.

Given the information above, we believe the best substrate sequence based on the peptide library we tested would be the sequence of VALPep 1. That is, an AcLys at position #1, Lys at position #2, and Ala at position #5. Based on our data, it seems the residues at Position #3 and #4 are not as important. Thus, provided our interest in developing PRMT1 inhibitors, we used our knowledge from previously reported studies identifying a peptide-based PRMT inhibitor that incorporates an active site Cys-modifying warhead in place of the guanidinium of the substrate Arg [23]. To that end, the guanidinium side chain of the Arg residues at position 6 in VALPep 1 was replaced with the chloroacetamidine warhead. This inhibitor was incubated with PRMT1 and the IC_50_ value was measured. Over the past decade, numerous small-molecule and peptide-based inhibitors targeting PRMT1 have been developed, demonstrating a range of potencies and selectivities. Peptide-based inhibitors exhibit IC_50_ values typically between 1 and 100 µM, whereas small-molecule inhibitors often show greater potency, with some reporting IC_50_ values as low as 0.0011 µM. These inhibitors differ in their mode of action, targeting either the arginine-binding site, the SAM binding site, or both sites as bisubstrate inhibitors [31,32]. The inhibitor reported herein, ValPep1 Warhead Peptide, demonstrates an IC_50_ value of 3.47 ± 0.46 µM, ranking among the most effective PRMT1 peptide inhibitors developed to date.

Ultimately, we can utilize the substrate specificity information garnered from this study regarding PRMT1 along with previous studies on the PRMTs to generate more potent and specific inhibitors for this enzyme family.

## 5. Conclusions

Utilizing a medium-throughput screening assay, we identified novel peptide substrates, which were utilized to identify key recognition elements that improved PRMT binding and catalysis. Our histone-derived library consisted of only a few amino acids in varying positions around the site of modification; however, we uncovered specific sequence features that significantly improved enzymatic efficiency. These included features such as long, uncharged amino acids at position #1, positively charged residues at position #2, and small non-polar residues adjacent to the substrate Arg. There was no preference observed at positions #3 and #4. While these findings do provide some insight into the substrate specificity of PRMT1, they also enabled us to design a potent, peptide-based inhibitor with a submicromolar IC_50_ value. In conclusion, this study offers a framework for the development of future PRMT1 inhibitors and serves as a model for screening larger, more diverse compound libraries aimed at uncovering substrate specificity and disease relevance across the PRMT enzyme family.

## Figures and Tables

**Table 1:** The “hit” wells and peptide sequences.

Well #	Variable Sequence
**B7**	AXKVV–Linker
**C1**	XKVAA–Linker
**C6**	KAXKK–Linker
**D5**	VKVCX–Linker
**F8**	XKCKV–Linker
**G2**	XCCVA–Linker
**G7**	XWWAA–Linker

Linker = RGGKGLGKGGAK.

**Table 2:** List of “Hit” and “Non−Hit” Peptide Sequences along with the Kinetic Parameters.

Peptide	Sequence	*k_cat_* (min^−1^)	*K_m_* (µM)	*k_cat_*/*K_m_* (M^−1^ min^−1^)
Well B10 Peptide	WWXXWRGGKGLGKGGAK	ND	ND	ND
Well E9 Peptide	VXWAWRGGKGLGKGGAK	ND	ND	ND
Well D5 Peptide	VKVCXRGGKGLGKGGAK	1.91 ± 0.113	6.78 ± 1.77	2.82 × 10^5^
Well G2 Peptide	XCCVARGGKGLGKGGAK	0.986 ± 0.004	25.4 ± 3.95	3.89 × 10^4^

The five variable amino acids, X = Acetylated Lysine, W = Tryptophan, C = Cysteine, V = Valine, are underlined. ND: The kinetic parameters of this peptide substrate were not determined due to low amount of product formation.

**Table 3:** List of Validation Peptide Sequences along with the Kinetic Parameters.

Peptide	Sequence	*k_cat_* (min^−1^)	*K_m_* (µM)	*k_cat_*/*K_m_* (M^−1^ min^−1^)
VALPep1	XKCKARGGKGLGKGGAK	2.46 ± 0.080	8.72 ± 1.20	2.82 × 10^5^
VALPep2	XKKKARGGKGLGKGGAK	2.58 ± 0.174	7.82 ± 2.23	3.29 × 10^5^
VALPep3	XKCVVRGGKGLGKGGAK	0.927 ± 0.036	6.92 ± 1.16	1.34 × 10^5^
VALPep4	XKKVVRGGKGLGKGGAK	1.61 ± 0.104	7.42 ± 2.02	2.16 × 10^5^
AcH4-16	SGRGKGGKGLGKGGAK	0.320 ± 0.001	170 ± 25.0	1.90 × 10^3^

The five variable amino acids, X = Acetylated Lysine, W = Tryptophan, C = Cysteine, V = Valine, are underlined.

## Data Availability

The original contributions presented in this study are included in the article/Supplementary Materials. Further inquiries can be directed to the corresponding author.

## Supplementary Materials

The following supporting information can be downloaded at: https://www.mdpi.com/article/10.3390/biom15111494/s1, Supplemental Table S1: Validation of Microwell Plate Peptide Masses; Supplemental Table S2: Validation of Individually Synthesized Peptide Masses; Supplemental Table S3: Sequences of the random peptides; Supplemental Figure S1: Kinetic parameters of “Hit” Peptides; Supplemental Figure S2: Kinetic parameters of “non-Hit” Peptides; Supplemental Figure S3: Kinetic parameters of Consensus Peptides, all Mass Spectra of Peptides.

## Abbreviations

The following abbreviations are used in this manuscript:

PRMT	Protein Arginine Methyltransferase
PTM	Post-translational Modification
EDTA	Ethylenediaminetetraacetic acid
BSA	Bovine Serum Albumin
