# Supplementary material for: The Identification of a Sub-Micromolar Peptide-Based Protein Arginine Methyltransferase 1 (PRMT1) Inhibitor from a Plate-Based Screening Assay
Source: Biomolecules. 2025 Oct 23;15(11):1494. doi: 10.3390/biom15111494 (PMC12650519; doi:10.3390/biom15111494)
Supplement: Supplementary file 1 [file biomolecules-15-01494-s001.zip › biomolecules-3912325-supplementary.pdf]

The Identification of a Sub-Micromolar Peptide-Based PRMT1 Inhibitor from a Plate-Based Screening Assay

Tina M. Sawatzky[1], Sarah A. Mann[1], Jordan Shauna Tucker[1], Aida A. Bibart[1], Corey P. Causey[1], Bryan Knuckley[1]*

[1]*Department of Chemistry & Biochemistry, University of North Florida, Jacksonville, FL, U.S.A*
**Corresponding Author*

Supplemental Information

Supplemental Table 1. Validation of Microwell Plate Peptide Masses

Peptide	Sequence	Expected	Observed
**Well C4 Peptide**	**WXVWKRGGKGLGKGGAKD**	**1969.99 (M+2H$^+$) = 984.99**	**985.68**
**Well C9 Peptide**	**AAXKCRGGKGLGKGGAKD**	**1743.85 (M+2H$^+$) = 871.93**	**871.46**
**Well D9 Peptide**	**CWVXKRGGKGLGKGGAKD**	**1886.93 (M+2H$^+$) = 943.47**	**944.89**
**Well E11 Peptide**	**KXAWKRGGKGLGKGGAKD**	**1883.97 (M+2H$^+$) = 941.99**	**942.90**
**Well G4 Peptide**	**KKAAWRGGKGLGKGGAKD**	**1783.99 (M+2H$^+$) = 891.99**	**893.06**
**Well G6 Peptide**	**KKAKCRGGKGLGKGGAKD**	**1757.99 (M+2H$^+$) = 879.95**	**880.36**

Supplemental Table 2. Validation of Individually Synthesized Peptide Masses

Peptide	Sequence	Expected	Observed
**VALPep1**	**XKCKARGGKGLGKGGAK**	**1685.01**	**1684.80**
**VALPep2**	**XKKKARGGKGLGKGGAK**	**1711.10**	**1710.94**
**VALPep3**	**XKCVVRGGKGLGKGGAK**	**1684.02**	**1684.74**
**VALPep4**	**XKKVVRGGKGLGKGGAK**	**1709.10**	**855.42**
**Well B10 Peptide**	**WWXXWRGGKGLGKGGAK**	**1986.39**<br>**$(M+2H^+)$ = 993.70**	**992.91**
**Well E9 Peptide**	**VXWAWRGGKGLGKGGAK**	**1798.16**<br>**$(M+2H^+)$ = 899.58**	**1798.15**
**Well D5 Peptide**	**VKVCXRGGKGLGKGGAK**	**1684.02**<br>**$(M+2H^+)$ = 842.51**	**842.69**
**Well G2 Peptide**	**XCCVARGGKGLGKGGAK**	**1632.01**<br>**$(M+2H^+)$ = 816.51**	**1631.25**
**VALPep1 Warhead Peptide**	**XKCKAXGGKGLGKGGAK**	**1718.96**<br>**$(M+2H^+)$ = 859.48**	**859.58**

Supplemental Table 3. Sequences of the random peptides.

	1	2	3	4	5	6	7	8	9	10	11	12
A	AKVAK	CK(Ac[K])AA	(Ac[K])WK(Ac[K])(Ac[K])	(Ac[K])AAKK	WA(Ac[K])WK	CACVK	CAAKA	A(Ac[K])ACK	CWVCK	KVW(Ac[K])(Ac[K])	KCAKK	(Ac[K])(Ac[K])ACV
B	KWA(Ac[K])W	KV(Ac[K])VA	KCAKV	AVAWK	KCWVV	CCC(Ac[K])C	VVK(Ac[K])A	A(Ac[K])VV(Ac[K])	(Ac[K])(Ac[K])AAV	W(Ac[K])(Ac[K])WW	WW(Ac[K])C(Ac[K])	VVCW(Ac[K])
C	AAVK(Ac[K])	WK(Ac[K])VV	CKKCC	KWV(Ac[K])W	W(Ac[K])ACW	KK(Ac[K])AK	WKA(Ac[K])(Ac[K])	CA(Ac[K])KC	CK(Ac[K])AA	CVCKK	KWW(Ac[K])(Ac[K])	AWAKA
D	CKKA(Ac[K])	KCAAA	KA(Ac[K])KV	CAAKK	(Ac[K])C(Ac[K])KV	KA(Ac[K])VV	AVWWW	(Ac[K])AVKK	K(Ac[K])VWC	AAV(Ac[K])W	VK(Ac[K])VA	W(Ac[K])KWC
E	(Ac[K])AA(Ac[K])W	A(Ac[K])CKK	(Ac[K])KC(Ac[K])C	WVCCC	VKAAC	A(Ac[K])(Ac[K])W(Ac[K])	K(Ac[K])(Ac[K])(Ac[K])V	AV(Ac[K])(Ac[K])K	WAW(Ac[K])V	VKKWK	KWA(Ac[K])K	(Ac[K])(Ac[K])ACV
F	(Ac[K])(Ac[K])KKC	WAAVK	KW(Ac[K])KW	VWAVV	AAKVA	CAA(Ac[K])(Ac[K])	CCKKA	VKCK(Ac[K])	CKVAA	(Ac[K])CWWA	AWKC(Ac[K])	KKWKA
G	KW(Ac[K])WW	AVCC(Ac[K])	(Ac[K])AW(Ac[K])(Ac[K])	WAAKK	VV(Ac[K])WK	CKAKK	AAWW(Ac[K])	KW(Ac[K])WK	ACA(Ac[K])K	AAA(Ac[K])K	KAAKW	(Ac[K])KVVW
H	CCVAV	WVKVA	(Ac[K])CAWA	AKVKA	W(Ac[K])CC(Ac[K])	VAWCC	CW(Ac[K])VV	K(Ac[K])WKA	KAKA(Ac[K])	GGRGG	GG-ADMA-GG	GGRGG

Variable sequence of 5 residues below were added to [R5R4R3R2R1]-R-GGKGLGKGGAK; Listed above as **R1R2R3R4R5**

Abbreviations are designated as **K** = Lysine, **A** = Alanine, **Ac[K]** = Acetylated Lysine, **W** = Tryptophan, **V** = Valine, **C** =Cysteine

Supplemental Table 3. Sequences of the random peptides

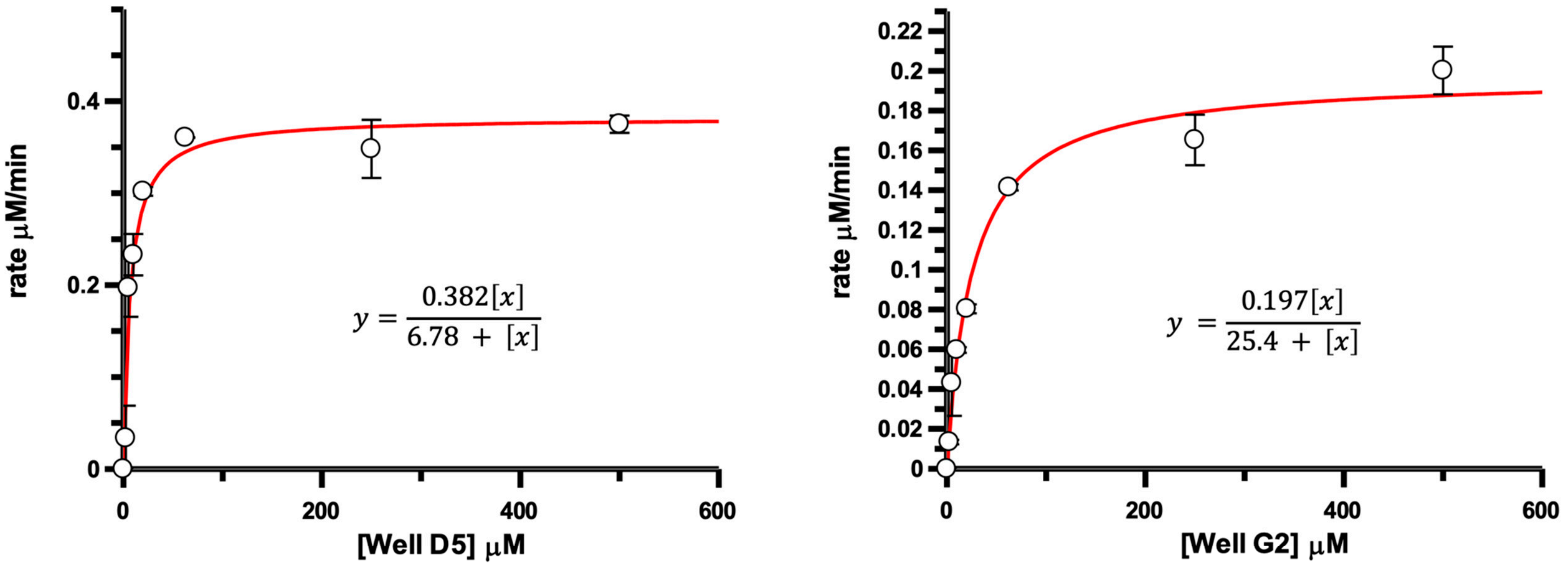

Supplemental Figure 1. Kinetic Parameters of "Hit" Peptides (A) Well D5 and (B) Well G2

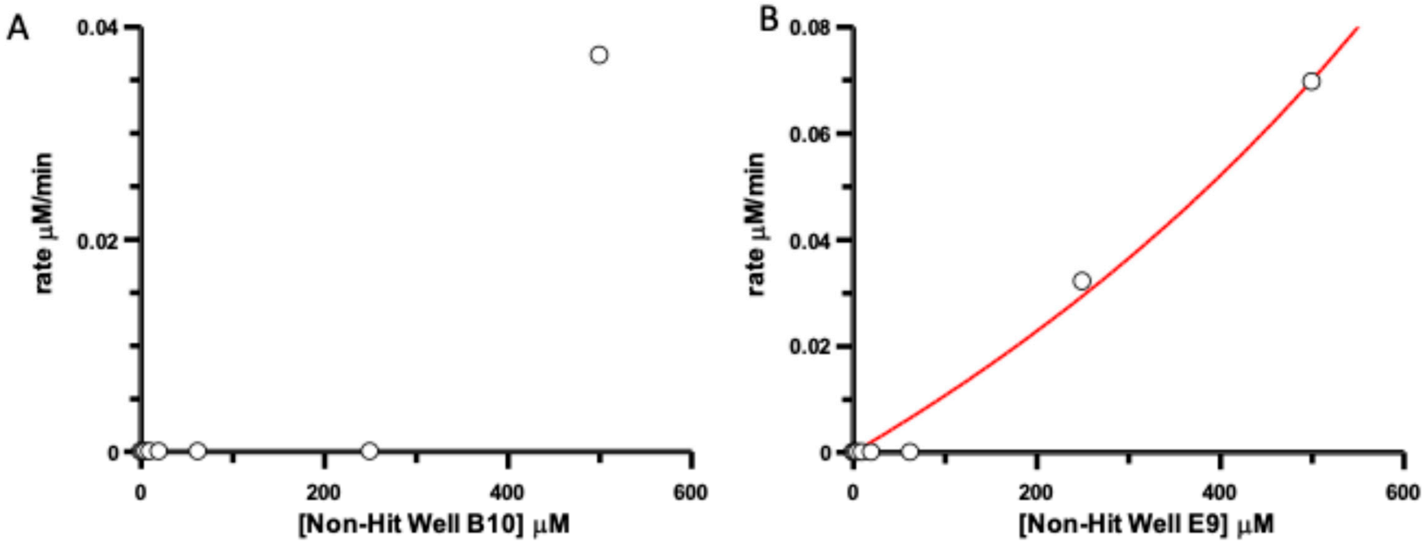

Supplemental Figure 2. Kinetic Parameters of "non-Hit" Peptides (A) Well B10 and (B) Well E9.

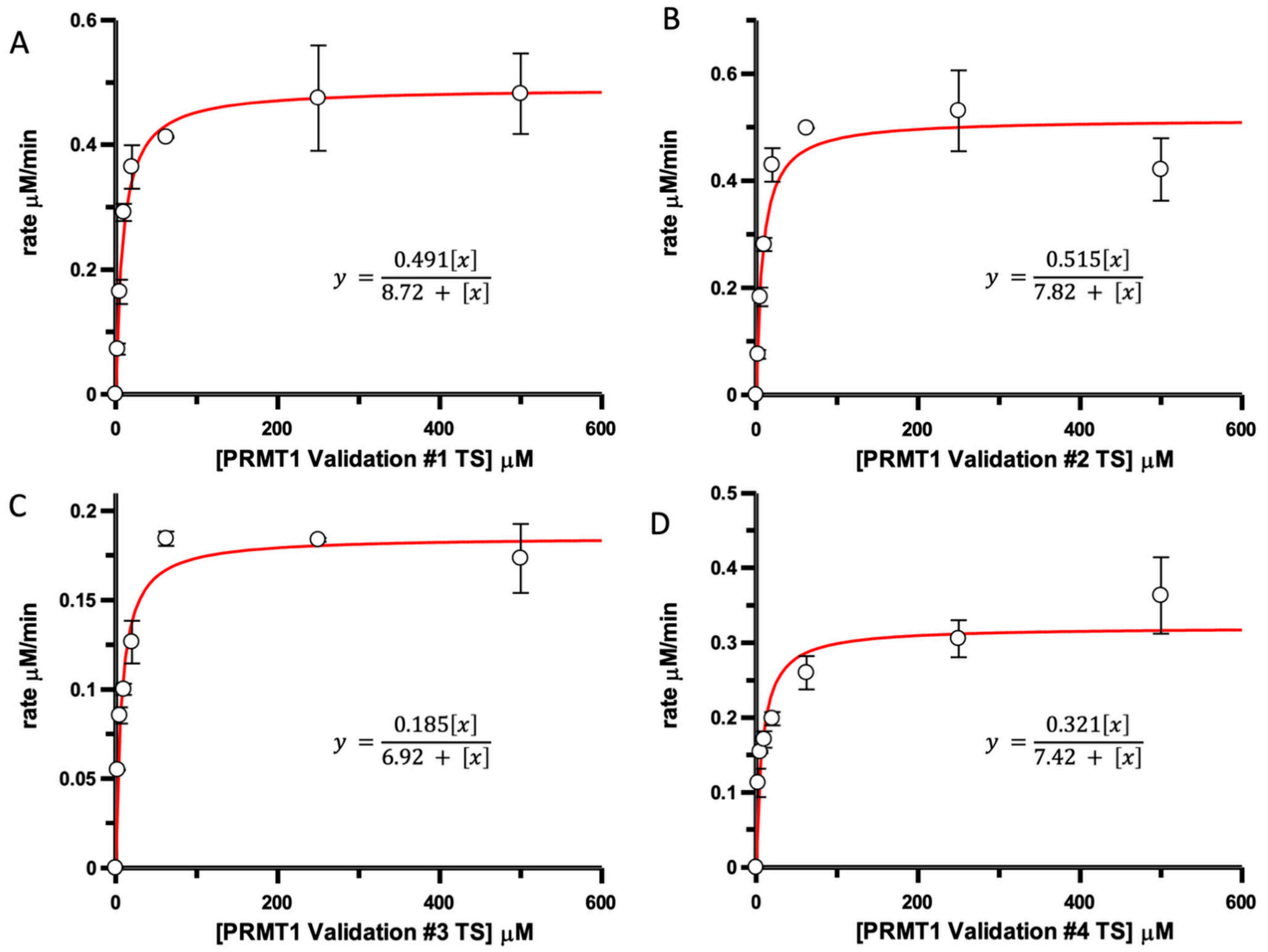

Supplemental Figure 3. Kinetic Parameters of Consensus Peptides, (A) VALPep1, (B) VALPep2, (C) VALPep3, (D) VALPep4
